# Life satisfaction in three Namibian cultures

**DOI:** 10.4102/ajopa.v8i0.205

**Published:** 2026-10-23

**Authors:** Christopher J. Hopwood, Gayoung Son, Elizabeth Shino, Michelle Rahm, Amber G. Thalmayer

**Affiliations:** 1 Department of Psychology, University of Zurich, Zurich, Switzerland; 2 Department of Psychology, University of Bern, Bern, Switzerland; 3 Department of Psychology, University of Namibia, Windhoek, Namibia

**Keywords:** satisfaction with life, culture, assessment, Namibia, Africa, well-being

## Abstract

Life satisfaction is one of the most studied variables in psychology, and researchers often compare broad cultural groups, but limited attention has been given to comparisons of sub-cultures within African nations. This study aimed to compare life satisfaction across three cultural groups in Namibia. We compared life satisfaction among participants within Namibia (*N* = 1912) who speak English, Khoekhoe or Oshiwambo. We first fit measurement models for life satisfaction in each group separately and then examined measurement invariance across samples. We then used invariant indicators to compare these groups in terms of mean levels and correlations with demographic variables and health. In general, evidence supported the validity of life satisfaction across groups. However, some interesting patterns emerged suggestive of cultural variation. For instance, life satisfaction was only significantly associated with age in the English sample and was only associated with health in the English and Khoekhoe samples. These findings support the assessment of life satisfaction in diverse Namibian cultures and point to variability in the factors that affect quality of life among different subgroups.

**Contribution:**

Findings are discussed in terms of cultural variation within Namibia and other African subgroups and regarding broader issues around understanding life satisfaction in majority world populations.

## Introduction

Life satisfaction, one of the most common constructs studied in psychology, refers to an individual’s global cognitive evaluation about how well their life is going (Diener et al., 1985). The Satisfaction With Life Scale (SWLS; Diener et al., 1985) was developed to provide a succinct, psychometrically sound measure life satisfaction, which is considered the cognitive component of a broader subjective well-being construct. Diener and colleagues initially generated a larger pool of items intended to reflect judgements about life quality, administered these to diverse samples and through exploratory and confirmatory factor analyses distilled the instrument to a unidimensional set of five items that best captured the construct without redundancy. The scale uses a seven-point Likert response format (ranging from ‘strongly disagree’ to ‘strongly agree’); higher scores indicate greater life satisfaction; sample items include statements such as ‘In most ways my life is close to my ideal’ and ‘If I could live my life over, I would change almost nothing’. These properties have helped make the SWLS one of the most widely used measures of life satisfaction in psychological research and applied settings globally. Since its introduction, the SWLS has been extensively used in research on well-being, quality of life and related fields, appearing in studies with diverse populations and age groups and translated into numerous languages. Meta-analytic and validation work has confirmed the scale’s generally high internal consistency, with Cronbach’s alpha coefficients typically in the 0.80s or above and acceptable test–retest reliability across intervals from weeks to years, indicating that the measure captures a relatively stable cognitive evaluation while remaining sensitive to life changes (Diener et al., 1985; Pavot & Diener, 1993). Confirmatory factor analyses across cultures and demographic groups overwhelmingly support a single underlying factor representing global life satisfaction although some investigations have explored alternative structures based on item content (e.g. present versus past orientation; Arrindell et al., 2022). Evidence for the SWLS’s validity is robust and multifaceted. Discriminant validity is demonstrated by its lower correlations with measures of affective intensity and unrelated constructs, indicating it assesses a distinct cognitive judgement rather than transient mood states (Arrindell et al., 1999; Pavot & Diener, 1993). Predictive validity has also been shown in longitudinal studies where SWLS scores relate meaningfully to future outcomes and life events (Pavot & Diener, 2009). Taken together, this body of research supports the SWLS as a reliable, valid and practical tool for assessing life satisfaction across a wide range of research and applied contexts. Life satisfaction, as measured by the SWLS, is not only partly heritable but also influenced by the environment (Hahn et al., 2013). Life satisfaction is relatively stable over the course of life, with declines often observed in old age (Baird et al., 2010), although this pattern may depend on issues such as how cohort and time effects are dealt with (De Ree & Alessie, 2011), what other variables are treated as controls or confounders (Bartram, 2021) and potentially contextual factors such as culture. Most studies have found that people who earn more money also tend to have higher levels of life satisfaction (Bai et al., 2021; Cheung & Lucas, 2015; Lucas et al., 2004; Vázquez et al., 2013), and some evidence also suggests a positive association between life satisfaction and level of education (Checa et al., 2019; Vázquez et al., 2013). Evidence for gender effects on life satisfaction is mixed, with some studies finding slightly higher levels among women (Joshanloo & Jovanović, 2020) and others finding no differences (Batz-Barbarich et al., 2018; Chen et al., 2020). There is robust evidence that life satisfaction is positively related to health (Arrindell et al., 1999; Diener et al., 2017; Diener & Chan, 2011; Ngamaba et al., 2017). Indeed, life satisfaction is so important that it is considered an outcome for comparing the effectiveness of social policies worldwide (Diener, 2000; Diener et al., 2015; Pavot & Diener, 2008). However, associations between life satisfaction and demographic or health variables may vary across cultures (Diener & Diener, 1995; Steptoe et al., 2015). A major limitation of existing research on life satisfaction is that it is predominantly informed by research in the approximately 11% of the world’s population living in Western industrialised contexts (Thalmayer et al., 2021b). In the ‘majority world’, that is, all the other contexts around the globe, this research is less developed. For example, of the 462 studies reported in a recent meta-analysis on life satisfaction (Anglim et al., 2020), 83% of the studies were from Western contexts, with only two from sub-Saharan Africa (0.4%: South Africa and Nigeria), six from Latin America and the Caribbean (1.3%), 13 from the Middle East (3%: Iran and Israel) and 42 from East and South Asia (9.1%). Existing evidence suggests that culture may impact how life satisfaction is conceptualised, experienced and expressed in ways that could impact measurement. Culture may also moderate associations between life satisfaction and demographic and health outcomes. Moreover, whereas most cross-cultural research has compared Western samples to majority world samples, much less attention has been paid to how cultural groups within the majority world differ from one another. There are 54 African countries and around 2000 African languages, a third of the world’s living languages (Hammarström, 2016). Thus, considerable ethnic and linguistic diversity exists within African countries, even though most studies that sample Africans typically focus on the nation as the cultural group. This diversity provides great potential for exploration of individual differences in human traits. In this study, we focus on life satisfaction within three cultural groups in Namibia, a diverse country on the southwestern coast of the continent.

### The Namibian context and three Namibian groups

Namibia is bordered by Angola, Zambia, Botswana and South Africa. The population in 2011 (Namibia Statistics Agency, 2013) was 57% rural, with a median age of 21 years, and an average age at first birth of 21.4 years. Adult literacy was 89%; 75% had completed either primary or secondary schooling and 6% had tertiary education. Unemployment was 37%, and about 50% of the adult population had income from wages or salaries. The largest economic sector (30%) was agriculture, fishing and forestry. In 1990, Namibia won independence from South Africa and transitioned from apartheid to a democratic society. Compared to many other African countries, Namibia has good civil liberty and political freedom (The World Bank, 2022). Despite this success, trauma from the decades of oppression and the fight for liberation remain (Bartholomew, 2016). The suicide rate is in the top quartile globally (World Health Organization, 2022), and many researchers have voiced concern that available psychological services are inadequate to meet public mental health needs (e.g. Bartholomew, 2017; Shifiona et al., 2006). In the face of these difficulties, reliably assessing life satisfaction could inform evidence-based policies for increasing resilience in society. Although English replaced Afrikaans as the sole official language of Namibia after independence in 1990, 10 national languages (Frydman, 2011) are recognised and are available in schools and at the university level. Of these, the two most spoken are Oshiwambo, spoken by nearly 50% of the population, and Khoekhoe, spoken by about 12% (Namibia Statistics Agency, 2013). These languages are totally unrelated, belonging to the Bantu and the Khoe-Kwadi language groups, respectively, and the groups that speak them have very different historical and cultural backgrounds (Hammarström, 2019; Witzlack-Makarevich & Nakagawa, 2019). The Ovambo people speak several dialects of Oshiwambo, a Bantu language that belongs to the larger Atlantic-Congo family. The group migrated from what is now the Zambia region into what is now northern Namibia and southern Angola around the 14th century. In contrast to many groups in Africa, their isolated, pastoral lifestyle meant that Ovambo people were less affected by Swahili-Arab and European traders or colonists, including the German colonisation of Namibia starting in 1884. Finnish missionaries arrived in the area in the late 19th century, and the Ovambo are now predominantly Lutheran Christians. The typical dress of contemporary Ovambo women, including a head scarf and long, loose dress, is also still based on the influence of those missionaries. Starting in the 1920s, contact increased through the German contract labour system (Likuwa & Shiweda, 2017). The South African government, which took over from Germany after World War 1, imposed apartheid in 1948, and independence was achieved in 1990. There are now about 2 million ethnic Ovambo, about half of whom live in the sandy, grassy high-altitude plains of northern Namibia and the other half in southern Angola. Seasonal rainfall floods the region into temporary lakes and islands, shaping the rhythm of Ovambo subsistence agriculture and herding. As the majority group in Namibia, the Ovambo lead the government. However, many, if not most, Ovambo maintain a rural lifestyle. This makes them especially culturally distant from the Western groups that are most commonly represented in psychological research. These differences may impact the way life satisfaction is expressed, which may in turn impact the psychometric properties of a measure like the SWLS among Oshiwambo speakers. Likewise, it is possible that life satisfaction is related to different outcomes. For instance, gender may be related to life satisfaction differently because the Oshiwambo are a matrilineal society, in which women can have specific familial leverage related to family names and inheritance. Khoekhoe (also referred to as Nama or Damara) is the most widely spoken of approximately 15 extant click languages of southern Africa commonly referred to as ‘Khoesan’ (Güldemann, 2008; Haacke, 2017), more properly as part of the Khoe-Kwadi Family (Witzlack-Makarevich & Nakagawa, 2019). According to Kim and colleagues (2014), the original speakers of Khoesan languages, southern African hunter-gatherers and pastoralists, constitute an early branch in human history. Ancestors of the group migrated into the area from East Africa before later groups split off from East African populations in the migrations that populated the globe. Some Khoesan groups retain aspects of their traditional hunting and gathering culture today (Kim et al., 2014), making their contemporary societies likely to be still shaped by this economic lifestyle. Two main groups in Namibia, with differing cultural and ethnic backgrounds, speak Khoekhoe today, in addition to it being the institutionalised language of choice for many smaller Khoesan groups. The Damara, who live more in the central and north-western part of Namibia north of Windhoek, were traditionally hunter-gatherers (later pastoralists) related genetically to Bantu speakers (Pickrell et al., 2012). The Nama, living generally in the southern part of the country, had larger clans, more elaborate political organisation and more emphasis on hierarchy and the role of chiefs (Barnard, 2008), which are still important in Nama culture today. These cultural norms may have implications for how life satisfaction is expressed and experienced. For instance, as a relatively egalitarian society, it is possible that personal income will be less important for members of the Khoekhoe population than the Oshiwambo or English samples. English has been the national language of Namibia since independence in 1990 and is the language of instruction in secondary schools throughout the country. However, it is much more commonly used in the capital city of Windhoek, the hub for government and business, where many ethnolinguistic groups come together. Its use in social and business contexts is less consistent in other areas, where Afrikaans and African languages are also often used. Given greater exposure to Western values and customs and the cultural importance of youth in Western cultures, it is possible that younger age may be more strongly related to life satisfaction among English speakers.

### Life satisfaction in Namibia

Relatively few prior studies have assessed life satisfaction in Namibia, and these were typically in small samples in specific populations. Swami et al. (2022) found strong evidence for metric invariance and good evidence for scalar invariance of the SWLS across 65 countries but did not include Namibia among the three sub-Saharan African countries they sampled. Akokuwebe et al. (2023) found that life satisfaction was positively associated with education and wealth among migrant workers in South Africa. In general, Namibian participants who report higher life satisfaction also report more adaptive scores on other measures of psychological functioning and well-being in Africa in general (Perstling & Rothmann, 2012) and Namibia in particular (Kalomo et al., 2022; Rodriques & Pieters, 2019). A previous study with the Khoekhoe subsample of the data we use in this study found that SWLS had generally positive correlations with adaptive personality features, although the psychometric properties of the SWLS were not examined (Thalmayer et al., 2021a). Some research suggests that some items of the SWLS may not translate well to the Namibian context, leading to poor model fit and weak item-factor loadings (e.g. Martin & Cooper, 2017; Rothmann, 2013; Schutte et al., 2021; Sovet et al., 2016; Van Rensburg & Mostert, 2023).

### Cross-cultural measurement considerations

Establishing measurement equivalence is typically considered a crucial first step for comparing cultures on psychological variables such as personality traits or life satisfaction (Byrne & Van de Vijver, 2010; Fischer & Karl, 2019; Putnick & Bornstein, 2016; Vandenberg & Lance, 2000). Establishing configural invariance ensures that the same items can be used to reflect higher-order constructs, establishing metric invariance ensures that correlations with external variables can be directly compared and establishing scalar invariance enables a direct comparison of mean scores across groups. However, previous research suggests that establishing measurement equivalence across cultures can be challenging (Emerson et al., 2017; Jovanović et al., 2022). Some have even argued that the requirement to establish measurement equivalence may constrain cross-cultural research, and that researchers should bypass this step in comparing psychological constructs across cultures (Gardiner et al., 2019). In this study, we take a middle and pragmatic position. As described below, we first try to identify measurement models for the SWLS within each cultural group. We next examine measurement equivalence, with the view that it would be ideal to establish an invariant measure to enable direct comparison. We were open to the possibility that measures would need to be trimmed to compare scales across cultures, or that in some cases comparisons would need to be made with caution if invariance could not be established. We also took the position that patterns of non-equivalence may be informative about cultural differences and thus were interested in these findings in their own right.

### Present study

The goal of this study was to examine life satisfaction in three Namibian cultural groups. We adopted a construct-validation approach in which theory is tested through a logical sequence of psychometric analyses (Loevinger, 1957). Firstly, we used confirmatory factor analyses to test whether life satisfaction can be regarded as a coherent construct in each of the three samples separately. The availability of an English-speaking sample is particularly useful for this stage of analysis, as it allowed us to rule out language or translation at least partially as an explanation for why the measurement models might not fit. Secondly, we used measurement invariance testing to determine whether the meaning of life satisfaction was the same across groups. It is possible that the SWLS items intercorrelate in such a way that suggests they reflect meaningful constructs, but that the meaning of these constructs is not the same across groups. Such findings would provide the foundation for hypotheses about cultural factors that could impact life satisfaction research. In contrast, evidence for measurement invariance would suggest that these constructs do carry the same meaning across cultures and would set the stage for comparing mean levels and associations. Thirdly, we examined associations between life satisfaction and (1) gender, (2) age, (3) education, (4) income and (5) health. Fourthly, we compared validity correlations across groups to determine whether individual differences in life satisfaction have the same implications across distinct Namibian cultures.

## Methods

This study uses data collected in three languages in Namibia in 2019. Ethical review clearance for this was granted by the University of Namibia Research Ethics Committee as part of a larger research project on mental health in Namibia. More details are described by Toscanelli et al. (2022).

### Participants

Three samples in Namibia were recruited, with sample sizes intended to exceed general guidelines for measurement invariance testing for survey-based studies (minimum 300 participants) to allow for complex analyses between multiple groups (Chen, 2007) and to account for the generally lower reliability of psychological surveys found in non-Western contexts (e.g. Schmitt et al., 2007; Thalmayer & Saucier, 2014), where survey research is less familiar. This was anticipated to be even more extreme, given our sampling of community and, in some cases, rural rather than student samples. A total of 2037 participants participated. Of these, 125 participants (6.54%) were excluded according to the predetermined criteria, as reported in Online Appendix 1 Table 1-OA1. The criteria were (1) surveys marked for exclusion by the interviewer due to a problem with the interview (*n* = 15); (2) any survey collected from a participant under age 18 years (*n* = 2); (3) surveys for which 15% of the responses were missing (*n* = 21) and (4) surveys for which there is almost no variation in responses or suspected random responding (*n* = 87). The final sample comprised 1912 participants. Khoekhoe speakers (*n* = 645) were recruited from towns and villages in north-western, eastern, southern and central Namibia in areas where the language is taught in schools. Oshiwambo speakers (*n* = 678) were recruited from northern Namibia in the regions of Oshana, Ohangwena, Oshikoto and Omusati, where use of the language predominates and is taught in schools. English speakers (*n* = 589) were recruited in Windhoek and surrounding areas in central Namibia, where English (the national language since independence in 1990) is more often spoken compared to less urban regions. Khoekhoe-speakers were aged 18–62 years (mean [*M*] = 34.8; standard deviation [s.d.] = 11.1), Oshiwambo-speakers 18–70 years (*M* = 33.3; s.d. = 12.1) and English-speakers 18–63 years (*M* = 31.7; s.d. = 9.6). A summary of other demographic details is provided in Table 1. Chi-square tests by Toscanelli and colleagues (2022) showed significant socio-demographic differences among the groups, in that income and educational attainment, including that of caregivers, were highest for English-speakers and lowest for Oshiwambo-speakers. The English-speakers were also most likely to have full-time or regular part-time employment, and Oshiwambo-speakers were least likely.

**TABLE 1:** Sample characteristics by group.

Demographic feature	Khoekhoe (*n* = 645)	Oshiwambo (*n* = 678)	English (*n* = 589)	Total (*n* = 1912)
Percent female	53	59	53	55
**Education level completed**				
Did not finish primary	4	10	1	5
Grade 7 primary	10	12	1	8
Grade 10 secondary	33	27	14	25
Grade 12 secondary	28	25	26	26
Vocational after Grade 10 or 12	5	8	5	6
Some university or diploma	13	11	1	13
University Bachelor’s degree	5	7	28	13
Master’s or post-graduate degree	1	2	9	4
**Employment status**				
Not currently working	37	26	14	26
Students	22	21	25	22
Work at home, other unpaid work	10	7	5	8
Seeking paid work	19	13	7	13
Occasional paid work	15	7	5	9
Regular part-time paid work	8	2	9	6
Regular full-time paid work	35	29	48	37
**Median monthly income**				
None	15	32	9	19
Between N$1.00 and N$500.00	26	24	17	22
Between N$500.00 and N$1500.00	16	17	11	15
Between N$1500.00 and N$3000.00	13	8	9	10
Between N$3000.00 and N$5000.00	9	6	6	7
Between N$5000.00 and N$10 000.00	11	6	17	11
Over N$10 000.00	9	8	32	16

Note: Values in percentages. Employment status columns sum to more than 100% because participants could be in more than one category. Income is reported in Namibian dollars.

### Procedure

Translations of the survey into Khoekhoe and the Oshikwanyama dialect of Oshiwambo were created using a multi-step process according to the guidelines of the World Health Organization (described in detail by Toscanelli et al., 2022; Online Appendix 1 Table 2-OA1). The English version of the interview was phrased in the first person, as these participants completed the paper-and-pencil version of the interview. The Oshiwambo and Khoekhoe versions were phrased in the second person, as interviews were conducted orally. Although Oshiwambo and Khoekhoe speakers communicate principally in these languages, schools often teach in Afrikaans or English, and individuals are less likely to be able to read or write in their language. Data collection efforts were coordinated with local leaders (e.g. constituency councillors) with the support of collaborators from the University of Namibia and local research assistants. Recruitment occurred in a 1–2-month period after an intensive training weekend, with on-the-ground support provided by the research team, including the second, third and last authors. Each language sample was recruited by teams of 8–15 interviewers. Interviewers conducted 30–100 approximately 40-min survey interviews each. Interviewers were either data collectors who had previous experience in collecting oral interviews or written surveys; teachers of the respective language in primary or secondary schools or psychology or sociology graduates from the University of Namibia. Interviewers approached participants at various locations, including their communities and neighbouring villages. They collected demographic information such as age, gender, physical health, income level, work status and the education level of the participant and their parents, as well as the location and general process of the interview (e.g. ‘participant was sincere’ or ‘took it as a joke’). All participants gave informed written consent before joining the study and received compensation of either a voucher for internet access, a gift of cans of fish (Oshiwambo speakers) or a gift bag including sugar, coffee, tea and powdered milk (Khoekhoe speakers).

### Measures

The SWLS (Diener et al., 1985) is a five-item instrument assessing global life satisfaction. It contains the following items: (1) ‘In most ways, my life is close to my ideal’; (2) ‘The conditions of my life are excellent’; (3) ‘I am satisfied with my life’; (4) ‘So far, I have gotten the important things I want in life’ and (5) ‘If I could live my life over, I would change almost nothing’. Participants indicate agreement on a 7-point Likert scale ranging from ‘strongly agree’ (7) to ‘strongly disagree’ (1). Reliability is described below. We measured health with a single item ‘In general, would you say your health is …’ with responses ranging from ‘excellent’ (7) to ‘poor’ (0). This item correlates strongly with longer and more in-depth measures of health status (Desalvo et al., 2006; Desalvo & Muntner, 2011).

### Analyses

The data were analysed using R 4.4.2 (R Core Team, 2022). Confirmatory factor analysis (CFA) and measurement invariance analyses, as well as computation of latent means and correlations, were carried out with the lavaan 0.6–12 (Rosseel, 2012), semTools 0.5–6 (Jorgensen et al., 2022) and psych 2.2.9 (R Core Team, 2022) packages. We first fit a unidimensional measurement model for the SWLS separately in each group. Distribution-free methods were adopted for CFA and SEM modelling using Weighted Least Squares Mean and Variance Adjusted (WLSMV) due to multivariate nonnormality. The comparative-fit index (CFI; Cheung & Rensvold, 2002) and root mean square error of approximation (RMSEA; Steiger, 1990) were used to evaluate model fit. Values of 0.95 or higher on CFI and values below 0.08 on RMSEA were interpreted as indicating good fit (Hu & Bentler, 1999). When necessary, we trimmed items with low path coefficients to improve model fit. However, we were biased in favour of retaining items across cultural groups to enable measurement invariance tests and thus were open to tolerating less than good fit within reason. If we could fit a measurement model in two or more groups, we next evaluated measurement invariance for each scale across the three groups (Vandenberg & Lance, 2000) using multi-group confirmatory factor analysis (MG CFA; Svetina et al., 2020). Three levels of invariance were sequentially tested (Bowen & Masa, 2015; Byrne & Van de Vijver, 2010; Fischer & Karl, 2019; Putnick & Bornstein, 2016). Firstly, the baseline model tested configural invariance without any constraints on parameters. Secondly, metric invariance was tested by constraining item loadings to be equal across groups. Thirdly, scalar invariance was inspected by constraining item intercepts. Changes in both CFI and RMSEA greater than 0.01 were interpreted as indicating a significant decrease in fit. We then examined mean differences across groups and correlations with gender, age, education, income and health. These analyses were conducted using two approaches. In the more liberal approach, we computed all of these effects using the SWLS full-scale and manifest scores. This provided complete information but potentially ignored instances of measurement non-equivalence. We also computed correlations with a latent life satisfaction variable for which metric invariance had been established and compared means for latent variables across groups when scalar invariance had been established. This more conservative approach provides potentially more trustworthy estimates. We used a type 1 error rate of 0.05 for statistical significance but focused our interpretation on patterns of effect size.

### Ethical considerations

Ethical clearance to conduct this study was obtained from the University of Namibia Research Ethics Committee (No. FHSS/474/2019).

## Results

The full SWLS fits the data reasonably well in the English and Khoekhoe groups. Item 5, ‘If I could live my life over again, I would change almost nothing’, however, had a near-zero loading in the Oshiwambo group and low loadings in the other two. The CFA model fit results for both 5- and 4-item versions can be found in Table 2, and path coefficients can be found in Online Appendix 1 Figure 1-OA1. Given the problematic loading for item 5, we focused on latent scores from the four-item versions but also computed manifest scores with the 4- and 5-item versions to illustrate how the psychometrically trimmed and full versions function. Means, standard deviations and reliability estimates for full and trimmed scales across all three groups are shown in Table 3. Note that reliability improved with the removal of item 5 in all three samples.

**TABLE 2:** Confirmatory factor analysis model fit results for a one-factor model with 4- or 5-item Satisfaction With Life Scale.

Group	Items	*χ* ^2^	*df*	RMSEA	90% CI	CFI
Khoekhoe	5	21.38	5	0.07	0.04, 0.10	0.99
	4	2.57	2	0.02	0.00, 0.08	1.00
Oshiwambo	5	43.14	5	0.12	0.08, 0.14	1.00
	4	24.83	2	0.13	0.09, 0.18	1.00
English	5	25.79	5	0.09	0.06, 0.12	0.99
	4	17.57	2	0.12	0.07, 0.17	0.99

CI, confidence interval; RMSEA, root mean square error of approximation; CFI, comparative-fit index; *df*, degree of freedom.

**TABLE 3:** Means, standard deviations, Cronbach’s alphas and McDonald’s omega with 4- or 5-item Satisfaction With Life Scale per language group.

SWLS scale	Khoekhoe (*n* = 645)				Oshiwambo (*n* = 678)				English (*n* = 589)			
	*M*	s.d.	*α*	*ω*	*M*	s.d.	*α*	*ω*	*M*	s.d.	*α*	*ω*
Five-item SWLS	3.58	1.38	0.75	0.67	3.64	1.36	0.73	0.67	4.42	1.20	0.75	0.67
Four-item SWLS	3.70	1.52	0.79	0.76	3.62	1.54	0.82	0.79	4.55	1.28	0.77	0.75
Latent four-item SWLS	2.75	1.00	-	-	2.74	1.00	-	-	4.82	1.00	-	-

Note: Standard deviations (s.d.) of latent SWLS scores are all 1.00, since we set the factor variance to one in our latent model specification.

SWLS, Satisfaction With Life Scale; s.d., standard deviation.

### Measurement invariance

We next examined measurement invariance for the trimmed SWLS. Metric and scalar invariance were achieved for trimmed scales across all groups (Online Appendix 1 Table 3-OA1). Measurement invariance was also generally achieved for gender, age (across groups aged 18–26 years, 27–39 years and 40–70 years) and education (across groups up to Grade 10, between Grade 10 and 12 and those with university training) groups, although evidence for scalar invariance across education groups was equivocal in the Khoekhoe and English samples (Online Appendix 1 Tables 4-OA1–6-OA1).

### Mean differences across groups

Model comparison tests using likelihood ratio tests were computed to investigate whether the latent means differ across language groups. To ensure scaling of the latent variables, we selected the first item as reference indicator for each model. As can be seen in Table 3, the English sample scored substantially higher than the other two groups. This pattern was also observed in the manifest 4- and 5-item versions of the scale.

### Correlations

We first examined whether correlations were moderated by group using Cochran’s *Q* tests. Significant differences were found regarding age, income and health (Table 4). Across all three language groups, more educated participants reported higher life satisfaction, and in no groups were gender differences observed. There was a positive association between age and life satisfaction among English speakers but not for the other groups. A positive association was observed for income across all three groups, but it was stronger in the English and Khoekhoe groups. Finally, health was positively related to life satisfaction in the English and Khoekhoe groups only. Very similar patterns were observed when correlations were estimated with manifest variables (see Online Appendix 1 Table 7-OA1).

**TABLE 4:** Latent correlations between life satisfaction scales and demographic variables and health.

Scale	Education	Age	Gender	Income	Health
**Life satisfaction**					
*p*	0.17	< 0.01	0.89	< 0.01	< 0.01
**Khoekhoe**					
*r*	**0.20**	0.05	0.00	**0.33**	**0.23**
95% CI	**0.15, 0.24**	−0.03, 0.12	−0.04, 0.05	**0.26, 0.40**	**0.16, 0.30**
**Oshiwambo**					
*r*	**0.20**	−0.04	0.00	**0.15**	0.07
95% CI	**0.15, 0.24**	−0.12, 0.03	−0.04, 0.05	**0.07, 0.22**	−0.01, 0.14
**English**					
*r*	**0.20**	**0.18**	0.00	**0.29**	**0.23**
95% CI	**0.15, 0.24**	**0.10, 0.26**	−0.04, 0.05	**0.22, 0.37**	**0.15, 0.31**

Note: Cochran’s Q tests were used to test significant differences between latent SWLS and demographic variables. *p*-values in the first-row report whether the correlations differ significantly across groups (*α* < 0.05). In cases where there are no group differences (education and gender), the correlation is constrained to equality across groups. For purpose of this analysis, education was recoded into years of education (e.g. ‘Grade 7 primary’ = 7). The variable gender is binary coded: Female = 0, male = 1. Bold values are significant (*p* < 0.05).

CI, confidence interval.

## Discussion

Psychological science needs to become more representative of the majority world to be a true science of human experience (e.g. Thalmayer et al., 2021b). It is also important to examine variation within groups in the majority world in general, and Africa in particular, to account for the region’s rich cultural diversity. Life satisfaction is among the most important and widely studied psychological variables, and the SWLS is the most widely employed measure life satisfaction across the globe. In this study, we focused on life satisfaction in three culturally distinct groups in Namibia. In general, patterns established in the west translated reasonably well to these groups. The measurement model for the SWLS fit reasonably well across groups, and scores were positively related to education, income and health, as seen in the west. These findings support the generalisability of findings regarding life satisfaction to Namibian groups. However, some issues highlight the need to consider how culture can affect the expression and measurement of life satisfaction. In particular, one item did not function well in Namibia, and it was notable that results for the Oshiwambo sample were the most different from the other two Namibian samples as well as from Western contexts. The Oshiwambo group is arguably the most culturally distant of the three Namibian groups relative to Western, industrialised populations (Thalmayer et al., 2021a), including many subsistence farmers, and all living far from the capital. Nearly 10% of the Oshiwambo group did not finish primary school, and 32% reported no income. In comparison, only 4% of Khoekhoe-speakers and less than 1% of English speakers reported educational attainment lower than primary school, and only 15% of Khoekhoe speakers and 9% of English speakers reported having no income.

### Measurement structure and invariance

The item ‘If I could live my life over again, I would change almost nothing’ did not load on the general factor in the Oshiwambo group and had weak loadings in the other two groups (Online Appendix 1 Figure 1-OA1). These findings converge with prior studies in Namibia (Rothmann, 2013), South Africa (Appiah et al., 2020) and other contexts that also reported lower factor loadings for the fifth item (Bai et al., 2021; Casas et al., 2012; Jovanović et al., 2022; Vázquez et al., 2013). Pavot and Diener (2009) noted that the fifth item shows weak convergence with the rest of the scale, suggesting that this item is more related to past life experiences, thus requiring a summary of one’s life as opposed to current life satisfaction. As further elaborated by Bai and colleagues (2010), the four items together possibly involve a horizontal or a temporal summation of current life satisfaction, which is less influenced by past experiences. Due to this distinction, it has been proposed that researchers exclude the fifth item if they are interested in the participant’s present satisfaction with life (Pavot & Diener, 2009). We would add to this that ‘living one’s life over’ is an idiomatic expression in English, which may pose a very odd existential prospect if you are not accustomed to the expression and its intended meaning. This made the item quite challenging to translate, and despite the time and effort spent to do so, the item remained strange for participants, requiring longer deliberation and leading to more questions to the interviewers, thus increasing many aspects of research ‘cost’ in addition to problematic item properties. This speaks to the importance of considering differences in Global Englishes: American terms, phrasing and expressions are not necessarily familiar, even in the other 60-some English-speaking countries around the world, a reality that is not well addressed yet in cross-cultural research (Melchers et al., 2019; Thalmayer et al., 2025). Having excluded this item, we found reasonably good support for an invariant unidimensional model. The reliability of the resulting four-item scale is comparable to those reported in previous studies in other nations (Vassar, 2007) and in Namibia (Perstling & Rothmann, 2012; Rodriques & Pieters, 2019; Rothmann, 2013). The one exception was a study by Martin and Cooper (2017) that sampled 88 younger Himba adults living in rural Namibia. Results of that study and the current report suggest that measurement properties may tend to degrade the more culturally distant the sample gets from those of Western industrialised societies in which the instrument was developed and validated (Vittersø et al., 2002). This should not be surprising and highlights the importance of emic (bottom-up, indigenous) approaches to assessment. This applies to both the kinds of indicators being assessed (i.e. content validity) and assessment method (e.g. questionnaires). It is noteworthy that the abbreviated version functioned well across diverse demographic samples in all three groups. This is particularly notable for age, given that scalar invariance was rarely achieved across ages in different cultural groups (Emerson et al., 2017; Jang et al., 2017). In contrast, previous studies are similar to ours in suggesting invariance for gender and education (e.g. Arrindell et al., 2022; Bai et al., 2021; Checa et al., 2019; Jovanović et al., 2022).

### Group differences and associations

The English-speakers in the Namibian capital, including members of many ethnolinguistic groups from across Namibia, had the highest SWLS scores in the study. According to the scoring guideline from Pavot and Diener (2008), the English group mean score for SWLS is in the average range, whereas the means of the Khoekhoe and Oshiwambo groups are indicative of below-average life satisfaction, compared to adult samples from Western populations. Participant characteristics may have had a role in these differences. For example, the Oshiwambo group reported lower educational and income levels, and most of these participants were recruited in rural areas. Khoekhoe participants were recruited from within, but mainly outside of the capital city in smaller towns and villages. Due to speaking English, the new national language, and to living in or near the capital, the English-speaking participants can be understood to have more access to higher education, better-paying jobs and better infrastructure, which could increase their life satisfaction. A post hoc test revealed that participants living in the capital (*M* = 4.05; s.d. = 1.68) were indeed significantly more satisfied than those living outside of it (*M* = 3.61; s.d. = 1.46); *t*(191.33) = 2.81, *p* = 0.006. Higher life satisfaction measured with the SWLS for urban versus rural groups has been previously observed in Namibia and South Africa (Koen et al., 2022; Roos et al., 2013; Wissing et al., 2012). In these studies, authors have suggested that lower life satisfaction observed in traditional community samples may be related to higher poverty and less infrastructure available for rural groups, which might explain the disparity observed in our subsample. Replicating prior study results (Bai et al., 2021; Checa et al., 2019; Ngamaba et al., 2017; Vázquez et al., 2013), we found that those who reported better health, higher income, higher education or full-time employment reported higher scores on the SWLS compared to their counterparts with lower health, less income, less education or unstable employment. In contrast, similar to previous research (see Pavot & Diener, 2009), we did not observe age or gender differences in SWLS scores in this study. Overall, effects were stronger in the English and Khoekhoe groups than the Oshiwambo sample. This suggests that the life circumstances associated with life satisfaction may be moderated by culture within the Namibian population. For example, Oshiwambo speakers came largely from rural northern villages, where people tend to live on traditional homesteads in areas of communal land ownership. Different priorities may shape their well-being; for example, their status and roles in the community may be less linked to education and stable employment, making these factors less relevant. Instead, communal values referred to as ‘ubuntu’ in the literature may mean that strength of social relationships, fulfilment of social and community obligations and spiritual beliefs and practices might be more meaningful and predictive of life satisfaction (Adjei, 2019; Rotzinger et al., 2025; Sodi et al., 2021; Uugwanga et al., 2025). Including measures more relevant to these factors in future studies could clarify what predicts better satisfaction in this and other Namibian groups.

### Limitations and future directions

The cross-sectional nature of the current study does not allow us to distinguish the relative stability of life satisfaction or its sensitivity to context across Namibian sub-populations. The data in Khoekhoe and Oshiwambo groups were collected by interview, whereas the English group completed the survey via a paper-and-pencil method, which might impact item responses and comparability across groups. Mean differences across these groups could be a measurement confound, if people report higher life satisfaction on questionnaire measures relative to interviews. Additionally, although we examined three language groups with participants from many parts of Namibia, this study did not reflect the full diversity of the country and was not strictly representative of the groups we studied.

### Conclusion

This is the first study to examine life satisfaction across three culturally and linguistically distinct groups within Namibia. We found that a four-item version of the SWLS effectively measures life satisfaction across all three groups after removing one idiomatically phrased item. This suggests that a modified version of the SWLS can be used to assess life satisfaction in cultural groups within Namibia and potentially in other comparable groups. The English-speaking group reported somewhat higher levels of life satisfaction than the Khoekhoe or Oshiwambo. The Oshiwambo group, who are the least similar to Western samples on which the SWLS was developed, showed the weakest links between life satisfaction and income and health. Overall, these results support the generalisability of life satisfaction and the SWLS to Namibia but suggest that culture shapes the relevant associations.
